# Mutational Pathways and Trade-Offs Between HisA and TrpF Functions: Implications for Evolution via Gene Duplication and Divergence

**DOI:** 10.3389/fmicb.2020.588235

**Published:** 2020-10-14

**Authors:** Erik Lundin, Joakim Näsvall, Dan I. Andersson

**Affiliations:** Department of Medical Biochemistry and Microbiology, Uppsala University, Uppsala, Sweden

**Keywords:** duplication, divergence, trade-off, HISA, TrpF, *Salmonella enterica*, bi-functional

## Abstract

When a new activity evolves by changes in a pre-existing enzyme this is likely to reduce the original activity, generating a functional trade-off. The properties of this trade-off will affect the continued evolution of both functions. If the trade-off is strong, gene duplication and subsequent divergence would be favored whereas if the trade-off is weak a bi-functional enzyme could evolve that performs both functions. We previously showed that when a bi-functional HisA enzyme was evolved under selection for both HisA and TrpF functions, evolution mainly proceeded via duplication-divergence and specialization, implying that the trade-off is strong between these two functions. Here, we examined this hypothesis by identifying the mutational pathways (i.e., the mutational landscape) in the *Salmonella enterica* HisA enzyme that conferred a TrpF-like activity, and examining the trade-offs between the original and new activity. For the HisA enzyme there are many different paths toward the new TrpF function, each with its own unique trade-off. A total of 16 single mutations resulted in HisA enzyme variants that acquired TrpF activity and only three of them maintained HisA activity. Twelve mutants were evolved further toward increased TrpF activity and during evolution toward improved TrpF activity the original HisA activity was completely lost in all lineages. We propose that, aside from various relevant ecological factors, two main genetic factors influence whether evolution of a new function proceeds via duplication – divergence (specialization) or by evolution of a generalist: (i) the relative mutation supply of the two pathways and (ii) the shape of the trade-off curve between the native and new function.

## Introduction

Adaptation to novel selective pressures may require evolution of new enzymatic functions. One way of achieving this is by selective improvement of a weak promiscuous secondary activity already present in an existing native enzyme (Copley, 2003; Khersonsky et al., 2006; Khersonsky and Tawfik, 2010; Andersson et al., 2015; Gupta, 2016). The rate of evolution and the paths available when evolving toward a new function are expected to be constrained by the potential loss of the original enzyme activity. One potential solution to the trade-off problem between new and old functions is the occurrence of a duplication – divergence pathway where a new function is free to evolve while maintaining the old function (O’Brien and Herschlag, 1999; Khersonsky et al., 2006; Khersonsky and Tawfik, 2010; Näsvall et al., 2012; Pandya et al., 2014; Andersson et al., 2015). The trade-off between gain of a new and loss of an old function will determine the timing and frequency of the required amplifications (Khersonsky et al., 2006; Soskine and Tawfik, 2010; Andersson et al., 2015). That is, if the trade-off is strong where every increase in the new activity significantly reduces the original one, maintenance of the original activity would require a gene duplication in order to retain a gene that encodes the original activity. On the other hand, if the loss in original activity is limited or absent adaption could occur without any need for gene amplification, and the enzyme can evolve into an efficient generalist with broad specificity.

Some previous studies have indicated that trade-offs are weak when selecting for a new function, such that very large gains in the new function are associated with limited loss of the old function (Aharoni et al., 2005; Khersonsky et al., 2006; Kaltenbach et al., 2016). Previous studies also suggest that the pathway toward new activity involves several steps of improvement of a generalist enzyme that can do both reactions at the same time while slowly losing the original activity over time when gaining new activity (Chelliserrykattil et al., 2001; Matsumura and Ellington, 2001; Aharoni et al., 2005; Khersonsky and Tawfik, 2010; Rockah-Shmuel and Tawfik, 2012; Guo et al., 2014; Devamani et al., 2016).

We previously showed in the HisA-TrpF model system that duplication-divergence and specialization appeared more common than evolution of a bi-functional generalist enzyme, indicating a strong trade-off (Näsvall et al., 2012). Here, we used the *Salmonella enterica* HisA enzyme to examine the nature of the trade-offs observed during evolution toward new TrpF activity. The native function of HisA is in L-histidine biosynthesis whereas the native function of TrpF is in L-tryptophan biosynthesis. The HisA and TrpF enzymes perform similar catalytic reactions, but on different substrates (Wierenga, 2001; Sterner and Höcker, 2005), and their respective reactions are known to be carried out by the single enzyme PriA in organisms lacking a TrpF enzyme (Barona-Gomez and Hodgson, 2003; Due et al., 2011). Our results suggest that there are several mutational paths toward the new TrpF function, but that in all of the recovered paths toward improved TrpF activity the original HisA activity is always lost. Thus, there exists a strong trade-off between these two functions, which would promote evolution of the new activity by duplication-divergence (Näsvall et al., 2012).

## Results and Discussion

### Experimental System

We chose the *S. enterica hisA* gene for this study based on the conditional essentiality (required for growth in minimal media lacking histidine) of the HisA enzyme in *S. enterica* and the fact that the (βα)_8_-barrel fold is very common among enzymes (Wierenga, 2001; Sterner and Höcker, 2005), potentially allowing us to generalize our results to other enzymes.

**FIGURE 1:**
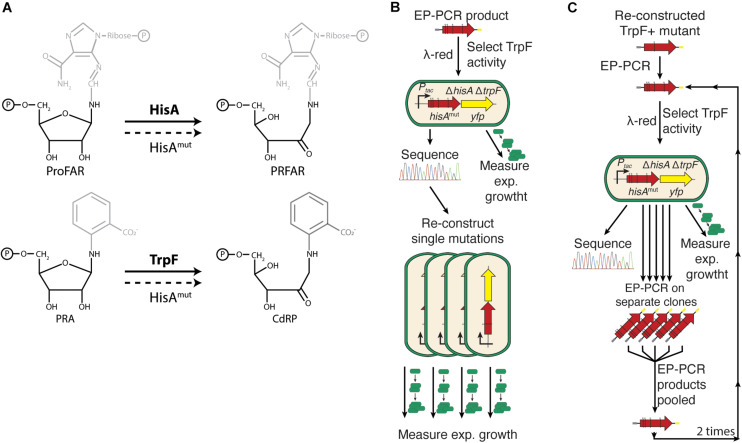
Experimental outline. **(A)** HisA and TrpF catalyze the same isomerization reaction but on two different phosphoribosyl compounds. The compounds share the phosphoribosylamine structure but differ in what chemical groups that are attached to the 1′-amino group. **(B)** After error-prone PCR to introduce random mutations in *hisA*, the gene was inserted into the chromosome, selecting for HisA mutants able to carry out the TrpF reaction. The DNA sequence was determined and the exponential growth rate in M9 minimal medium was used as an indirect measurement of the total cellular enzymatic activity. **(C)** Twelve single amino acid mutations were chosen for further evolution toward increased TrpF activity. Error-prone PCR was performed on these clones and selection for TrpF activity and analysis of DNA sequence and total cellular activity was as described under Figure 2B. The procedure was repeated two more times resulting in 1st step, 2nd step, and 3rd step improved clones (see Figures 2, 3).

The isomerases HisA and TrpF perform similar catalytic reactions, Amadori rearrangements of amino aldoses into amino ketoses, on substrates that share the same base sugar (5′-phosphoribosyl amines) to which different side-chains are attached (Figure 1A). HisA catalyzes the fourth step in the L-histidine biosynthesis pathway, the rearrangement of ProFAR (N′-[(5′-phosphoribosyl)formimino]-5-aminoimidazole-4-carboxamide ribonucleotide) to PRFAR (N′-[(5′-phosphoribulosyl) formimino]-5-aminoimidazole-4-carboxamide-ribonucleotide). TrpF catalyzes the third step in the L-tryptophan biosynthesis pathway, converting PRA (N′-(5′-phosphoribosyl)-anthranilate) to CdRP (1-[(2-carboxyphenyl)amino]-1-deoxyribulose 5-phosphate; Alifano et al., 1996; Henn-Sax et al., 2002; Figure 1A). Furthermore, the ability of HisA to acquire TrpF activity has previously been described (Jürgens et al., 2000; Näsvall et al., 2012). Additionally, there is also a naturally occurring HisA generalist enzyme (PriA) that catalyzes both reactions in bacteria lacking TrpF (Barona-Gomez and Hodgson, 2003; Due et al., 2011). An evolutionary connection between the two enzymes has also been suggested (Henn-Sax et al., 2002).

The functions of both HisA and TrpF are essential for growth on minimal medium. This allowed us to find mutations that introduce the new function (TrpF activity) by plating cells lacking the native TrpF enzyme on medium lacking tryptophan to select mutants able to overcome tryptophan auxotrophy (Näsvall et al., 2012). Additionally, as shown previously, by expressing HisA from a constitutive promoter, at levels where the growth rate of cells is limited by the rate of histidine or tryptophan synthesis, allowed the use of bacterial growth rates as a proxy of the total cellular HisA or TrpF activities. Throughout the text, *activity* designates the product of intracellular enzyme level and enzyme specific activity (Lundin et al., 2018). Thus, mutations that improve total cellular TrpF activity lead to faster growth in medium lacking tryptophan and mutations that reduce the total cellular HisA activity cause slower growth in medium lacking histidine, although we do not know if the relationship between activity and growth rate is linear or non-linear. As shown in a previous study, the dynamic range of using relative growth rate as a proxy for activity is considerable (Newton et al., 2017). Thus, a 300-fold reduction in HisA activity (caused by a combined reduction in enzyme level and specific activity) was detectable as a 6-fold reduction of relative growth rate (from the wild type relative growth rate set to 1.0 down to 0.16). Importantly, using relative growth rate as a proxy for activity allowed us to assess hundreds of enzyme variants, which would be impractical using biochemical assays.

To identify mutant HisA enzymes that acquired TrpF activity, the *hisA* gene was subjected to random mutagenesis by error-prone PCR in a strain with the native *trpF* gene deleted. The mutagenized gene was introduced into the *S. enterica* chromosome at the neutral *cobA* locus (i.e., insertions in the locus do not affect growth under the conditions tested), and expressed from a strong constitutive promoter. Subsequently, we selected for gain of TrpF activity by plating cells carrying a mutagenized *hisA* gene on minimal glucose medium plates lacking tryptophan.

### HisA Function Trade-Offs

**FIGURE 2:**
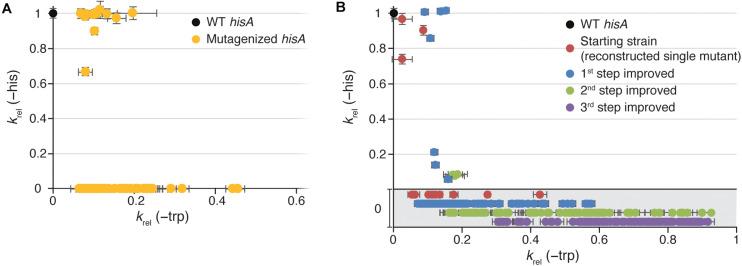
Growth rates of HisA mutant strains. Relative growth rates (*k*_*rel*_) in medium lacking histidine (*Y*-axis; as a proxy for total cellular HisA activity) or in medium lacking tryptophan (*X*-axis; as a proxy for total cellular TrpF activity). Error bars represent the standard deviation of eight replicates. **(A)** Characterization of all 74 TrpF-positive clones selected after mutagenesis of wild-type *hisA*. **(B)** Characterization of reconstructed TrpF-positive single mutants (red circles) and 1st step (blue), 2nd step (green), and 3rd step (purple) improved mutants. In the gray area, growth in medium lacking histidine was undetectable, and the data points are only separated along the *Y*-axis for visualization purposes.

To allow detection of mutants with low TrpF activity, we first expressed HisA from a strong promoter (*P*_*tac*_; Figure 1B). In total, 2.2×10^4^ *hisA* mutants were screened for TrpF activity. The HisA and TrpF activities of the mutants were assessed indirectly by measuring the growth rate under conditions when either HisA activity was limiting for growth (His absent and Trp present in growth medium) or when TrpF activity was growth limiting (His present and Trp absent in growth medium). Out of these, 73 clones had acquired TrpF activity, where 62 had lost all HisA activity and 11 retained some HisA activity (Figure 2A and Supplementary Tables S1, S2). This suggests that complete loss of HisA activity is frequent during evolution toward increased TrpF activity.

Among the 11 clones that retained the HisA activity, the TrpF activity varied extensively with relative growth rates (*k*_*rel*_) ranging from 0.07 to 0.44 as compared to 1.0 for a cell with a native *trpF* gene (Figure 2A and Supplementary Table S3). Nine out of these 11 strains showed a HisA activity similar to the wild-type and only two had a significantly lowered HisA activity. In a previous study, we identified two different HisA variants with TrpF activity and both variants were lacking HisA activity (Näsvall et al., 2012). One variant had the single amino acid substitution L169R and one variant had a duplication of amino acids 13-15 (“dup13-15”). The dup13-15 was shown to be able to catalyze both reactions when combined with either D10G or G11D, resulting in a bifunctional HisA-TrpF generalist enzyme.

As several of the isolated mutants in this study carried more than one mutation, *hisA* mutants encoding HisA enzymes with a single amino acid substitution were constructed to determine which changes conferred TrpF activity. Fourteen unique single mutations were found to give TrpF activity, out of which three retained their HisA activity (Table 1 and Supplementary Table S2). In addition, a mutation accidently introduced during reconstruction of one mutant lead to the isolation of an additional single amino acid substitution variant showing TrpF activity. Taken together with previously reported mutants, a total of 16 different mutations providing TrpF activity were identified. For 13 out of these 16 mutants, all HisA activity was lost, leaving three mutations that generated a bifunctional HisA enzyme (Table 1 and Supplementary Table S1).

**TABLE 1:** Amino acid changes providing TrpF activity.

Amino acid change	Nr. obs.	HisA activity	TrpF activity	Evolved further
dup13-15^1^	–	–	Yes	Yes
R15S	2	Yes	Yes	Yes
Q18R	11	Yes	Yes	Yes
G19R	9	–	Yes	Yes
D26V	1	–	Yes	–
G79R	1	–	Yes	–
A127G	1	Yes	Yes	Yes
D129A	4	–	Yes	Yes
D129G	9	–	Yes	Yes
D129P^2^	–	–	Yes	Yes
D129Y	3	–	Yes	–
G144R	1	–	Yes	–
W145R	4	–	Yes	Yes
L169R	10	–	Yes	Yes
D176A	1	–	Yes	Yes
D176V	18	–	Yes	Yes

*Sixteen different changes in HisA were shown to provide TrpF activity. 12 of these were chosen for further evolution toward increased TrpF activity. ^1^Re-constructed based on Näsvall et al. (2012). ^2^Isolated during re-construction of D129A. Nr. obs. = number of times the mutation occurred in the dataset.*

These findings show that the enzymatic specificity of HisA can be changed from converting ProFAR to PRFAR (HisA activity) into exclusively converting PRA to CdRP (TrpF activity) by at least 13 different single mutations causing amino acid substitutions. Furthermore, three different mutations at three different positions resulted in a promiscuous enzyme that was able to catalyze both reactions to some extent. As can be seen in Figure 2A, the trade-offs between the HisA and TrpF activities are overall very strong. Furthermore, these results suggest that there is considerable hidden potential for emergence of new activities in HisA. The HisA enzyme consists of 245 amino acids yielding 245 × 19 potential single amino acid substitutions. It is reasonable to assume that most of the 2205 single nucleotide substitutions were present in our libraries, but that very few mutants contained multiple substitutions in a single codon (and no other mutations). If all single amino acid substitutions that are accessible by single nucleotide changes in the *hisA* gene were present, our library contained 1457 unique single amino acid substitutions. Of these, about 1% (15/1457) could confer TrpF function. This suggests that there are many different pathways toward obtaining a new activity within HisA, illustrating the versatility of the (βα)_8_-barrel fold to acquire novel activities (Jespersen et al., 1993; Fong et al., 2000; Wierenga, 2001; Henn-Sax et al., 2002; Schmidt et al., 2003; Vega et al., 2003; Sterner and Höcker, 2005; Li et al., 2008; Evran et al., 2012).

### Evolution Toward Further Increased TrpF Activity

To determine if and how the shape of the trade-off curve depended on the starting strain, HisA was evolved toward increased TrpF activity in three steps (Figure 2B). Twelve of the 16 mutants with some TrpF activity were chosen as starting strains for the continued evolution toward higher TrpF activity (henceforth referred to as 12 lineages). Error prone PCR (EP-PCR) was performed on the *hisA* gene from all starting lineages separately. The PCR products were transformed into a *hisA*- and *trpF*-deleted strain and the cells were plated on selective media where the largest colonies were picked (designated 1st step improved clones). The procedure was repeated additionally two times resulting in mutants designated 2nd step and 3rd step improved clones, respectively. For each step and lineage, approximately 1 × 10^4^ clones were screened.

**FIGURE 3:**
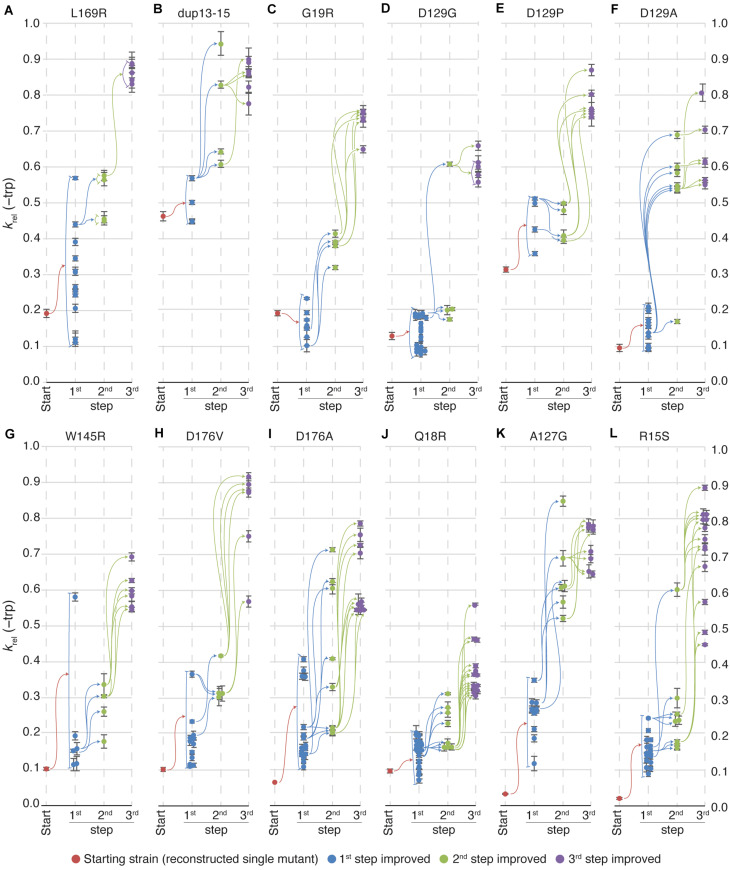
Relative TrpF activity in evolved clones. Growth rates of all lineages in medium lacking tryptophan were, on average, improved at each step of evolution. Certain lineages reached higher growth rates than others but there was no correlation between the growth rate of the starting clones and the 3rd step improved clones in the same lineages. Each vertical line represents a step in the evolution toward increased activity. Red dots indicate the starting clone for each lineage, blue dots represent 1st step improved clones, green represent 2nd step improved clones and, purple represent 3rd step improved clones. Colored arrows indicate that the clone to the right is a mutated version of the clone to the left (i.e., all mutations in the clone to the left are present in the clone to the right). Error bars represent the standard deviation of eight replicates. The starting mutations in the 12 lineages evolved toward increased activity were **(A)** L169R, **(B)** dup13-15, **(C)** G19R, **(D)** D129G, **(E)** D129P, **(F)** D129A, **(G)** W145R, **(H)** D176V, **(I)** D176A, **(J)** Q18R, **(K)** A127G, and **(L)** R15S. This strain was viable and formed colonies in 2 weeks on plates lacking tryptophan, but grew too slowly to observe growth in liquid media. Therefore, the relative growth rate for the single R15S mutant was plotted as 0.025.

In the majority of cases, the TrpF activity increased for each round of mutagenesis (as measured by growth rates in absence of Trp) and selection (Figure 2B and Supplementary Figure S1), but the relative increase at each step as compared to the lineage ancestor (Supplementary Figure S1A) and the maximum activity reached (Figure 2B and Supplementary Figure S1B) differed between the lineages. All lineages except dup13-15 and D129P started at an activity of *k*_*rel*_ = 0.03 to 0.2, and the 1st step improved clones showed a distribution range from 0.1 to 0.2 with a few outliers (Figures 3A–L), indicating that very few mutations allow for a rapid increase in TrpF activity in a single step. The TrpF activity of the 2nd step improved clones varied greatly, regardless of the lineage starting activity among the 1st step improved clones (Figures 3A–L and Supplementary Figure S1A). Among the 3rd step improved clones, the TrpF activity was on average further improved, with less variation than among 2nd step improved clones (Figures 3A–L and Supplementary Figure S1A).

To test the repeatability of these evolutionary pathways, we repeated the selection of improved clones in the L169R, dup-13-15, D129G and D176V lineages (Supplementary Figures S2A–D). For the L169R and dup-13-15 lineages, the 2nd step and 3rd step improvement steps were repeated (with the 3rd step based on the new clones from the 2nd step). For the new set of improved clones, the same pattern was observed as for the original set of improved clones (Supplementary Figures S2A,B compared to Figures 3A,B). Most 2nd step clones grew faster than all 1st step improved clones and similarly the 3rd step clones showed further increased TrpF activity as compared to the 2nd step clones.

For the D129G lineages and D176V lineages, the 3rd step improvement step was repeated (Supplementary Figures S2C,D). In the first set of transformations, a single 2nd step improved clone (the fittest clone) gave rise to all 3rd step improved clones in the D129G lineage (Figure 3D). Upon repeating this step, the same clone gave rise to faster growing clones, but also three other 2nd step improved clones gave rise to 3rd step improved clones–some of which had higher TrpF activity than any clone based on the fittest 2nd step improved clone. This demonstrates that the there is a large pool of mutations for improved activity, and that our screening may not have discovered all possible improving mutations.

It is notable that at least some additional mutations beyond the first recovered reduce fitness (Figures 3A,C,D,J). The reason for this is unclear but one possible explanation is that the *hisA* gene was amplified during the prolonged selection on plates, allowing the cells to express more enzyme and thus grow faster and form larger colonies. Before measuring growth rates of the mutants, the cells were grown in the presence of tryptophan which could have allowed unstable amplifications to segregate, thereby reducing fitness.

During evolution toward increased TrpF activity from the 12 chosen starting strains, HisA activity was successively lost. None of the variants evolved from the starting mutants lacking HisA activity regained HisA activity. More strikingly, in each step of improving TrpF activity in the lineages starting with any of the three mutants that retained HisA activity, a majority of the recovered clones lost their HisA activity and none among the 3rd step improved clones retained any HisA activity. This complete loss of HisA activity is in contrast to what is observed when random mutations (without any demand for acquiring TrpF activity) are introduced into HisA. Under such conditions, HisA appears relatively robust and very few single mutations abolish its activity (Lundin et al., 2018). Thus, the loss of HisA activity is associated with the gain of TrpF activity and not a consequence of mutation accumulation *per se*. A possible caveat is that our method of selection favored the mutations that caused the largest improvements in TrpF activity, and we may have missed mutants that caused such small improvements that they did not generate visibly larger colonies.

### The Shape of the Trade-Off Curve Is Dependent on *hisA* Expression Levels

A previous analysis of the distribution of fitness effects (DFE) of randomly introduced mutations in HisA showed that the effect of any mutation on HisA activity (measured as growth rate) is highly dependent on the HisA expression level, i.e., to what extent HisA is rate-limiting for growth (Lundin et al., 2018). The observed growth rate will be dependent on the total enzyme activity, which is a function of both its specific activity (kinetic efficiency, *k_*cat*_/K_*m*_*) and enzyme abundance in the cell (Soskine and Tawfik, 2010; Lundin et al., 2018). If the total activity of the enzyme exceeds the requirements for rapid growth, mutations will only reduce fitness if the total activity drops below the threshold required for rapid growth. In contrast, at high expression levels, the total activity is well above this threshold and mutations reducing protein activity might therefore not reduce organism fitness.

To examine the effect of expression levels, we placed all re-constructed single amino acid variants of *hisA* as well as the dup13-15 variant under the control of an arabinose inducible promoter and measured HisA and TrpF activity at high and low arabinose concentrations and compared these results with data obtained with the *P*_*tac*_ promoter. The high arabinose condition was chosen for maximum expression from the *P*_*araBAD*_ promoter and the low arabinose condition for the lower expression level to strongly limit growth by the HisA activity. The low expression level was chosen such that a strain with a wild-type copy of *hisA* showed a growth rate that was 72% of what was observed under high expression (Lundin et al., 2018). At this lower expression level, any mutational change that reduces the activity of the protein (reduction in the kinetic efficiency and/or concentration) will decrease organism fitness. The level of expression was assessed at all three conditions (*P*_*tac*_, *P*_*araBAD*_ high, *P*_*araBAD*_ low) by measuring Rfp expression from a *hisA-rfp* translational fusion (Supplementary Figure S4).

As shown in Supplementary Figure S5, the trade-offs between HisA and TrpF activities vary considerably depending on the specific mutation. Furthermore, the fitness effects of the mutations depend strongly on expression level, and at high expression level the deleterious effects of the mutations can often be partly buffered. This also suggests that an enzyme, whose expression can be up-regulated in response to a mutation that introduces a novel activity at the expense of an old one, is less restricted in evolving the new function.

### Restoring HisA Activity

Wild-type HisA does not have any detectable TrpF activity and here we show that 13/16 mutations resulting in TrpF activity will result in the complete loss of the original HisA activity. A strong trade-off situation, where all original activity is lost when gaining the new activity, is not accessible if there is a need to maintain the original activity (a reasonable assumption for natural systems). To examine if the original HisA activity can be restored while maintaining TrpF activity, we used nine HisA mutants that had acquired TrpF activity, but lost its original HisA activity, and selected for mutants with restored HisA activity. Clones with restored HisA activity were isolated from all lineages, but in most lineages the mutation enabling HisA activity was a reversion to the wild-type amino acid. However, in four lineages (in dup13-15, D129A, D129G, and L169R), we isolated mutant variants that did not revert back to the wild-type amino acid (Supplementary Table S4). In the dup13-15 lineage, all clones had a G11D mutation which we have previously found to restore HisA activity while still providing TrpF activity (Näsvall et al., 2012). The D129A and D129G lineages lost all TrpF activity when re-gaining HisA activity by a second mutation in a different position (A127E; Supplementary Table S4). In the L169R lineage, a L169Q mutation was found to restore HisA activity while losing all TrpF activity (Näsvall et al., 2012). Overall, the trade-offs appear reciprocal such that acquiring a new function eliminates the original and vice versa.

### Structural Explanations for Effects of Mutations

**FIGURE 4:**
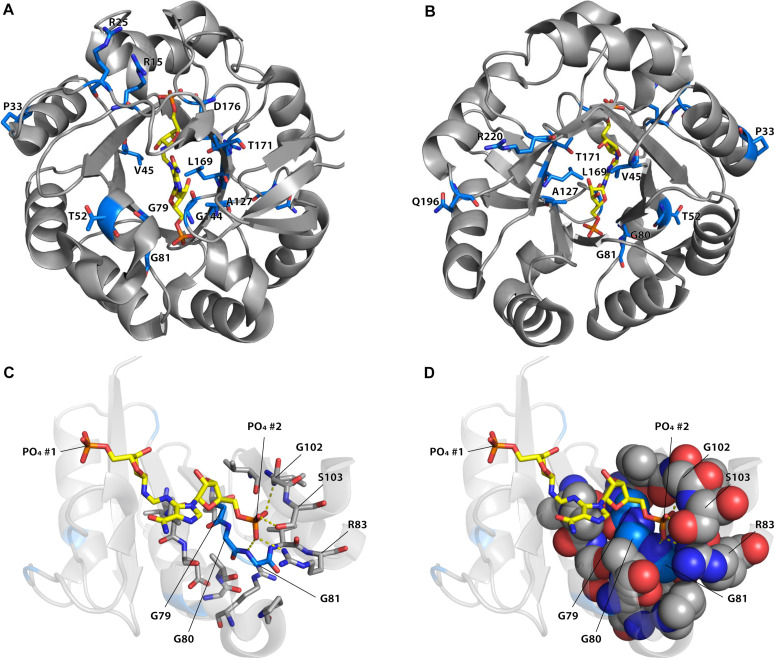
Positions of reoccurring mutations improving or inducing TrpF activity in HisA. **(A)** View from the catalytic face. **(B)** View from the face opposite to the catalytic face. The positions of the 16 mutations (in 15 positions) that was found more than five times in the dataset are represented as sticks with blue carbon atoms and with the identity of wild-type residue indicated. The native substrate of HisA (ProFAR) is shown with carbons in yellow color. **(C,D)** Positions of mutations in the phosphate 2 binding site. Only amino acids 1–106 are shown. Coloring is as in panels **(A,B)**. Residues containing atoms within 4 Å from G79, G80, G81, or phosphate 2 of ProFAR are indicated as sticks (in panel **C**) or as spheres (in panel **D**). The figure was generated using PyMOL (Schrödinger, 2015) from the crystal structure of *S. enterica* HisA D7N D176A with bound ProFAR (pdb id: 5a5w; Söderholm et al., 2015).

Among the different mutations that increased TrpF activity some occurred more frequently and were repeatedly found in the different lineages (Supplementary Figures S6–S8 and Supplementary Tables S5–S8). As can be seen in Figure 4, the most common mutations were located in or near the active site. For example, mutations in positions 80, 81, 83, and 102, which interact with the phosphate adjoining the non-reacting ribose (phosphate 2) of ProFAR (Söderholm et al., 2015) were common, as were mutations in the nearby positions 79, 106, and 107. Without structures and enzyme kinetic data of the different mutant proteins it is challenging to explain how they can confer their effect but some observations can be made. For example, the phosphate 2 binding site (Figures 4C,D) is likely to be important for proper positioning of the native substrate ProFAR, and mutations in nearby positions could alter the phosphate binding site to allow PRA binding (Newton et al., 2017). Purified wt HisA coordinates PO_4_^2–^ or SO_4_^2–^ in both phosphate binding sites (Söderholm et al., 2015), and the presence of a negatively charged ion in this site causes electrostatic repulsion of the negatively charged PRA, thus preventing binding of PRA and TrpF activity. Mutations affecting the phosphate 2 binding site and reducing the binding of PO_4_^2–^ and SO_4_^2–^ would likely increase binding of PRA, improving TrpF activity with a collateral loss of HisA activity. Other frequent mutations were found at position 15, which is located in a flexible loop (loop 1) of HisA that has an important role in interacting with the PRA substrate (Söderholm et al., 2015; Newton et al., 2017).

### The Biochemical Trade-Off Between Old and New Functions Influences Whether Evolution of a New Function Proceeds via Duplication – Divergence (Specialization) or by Evolution of a Generalist

**FIGURE 5:**
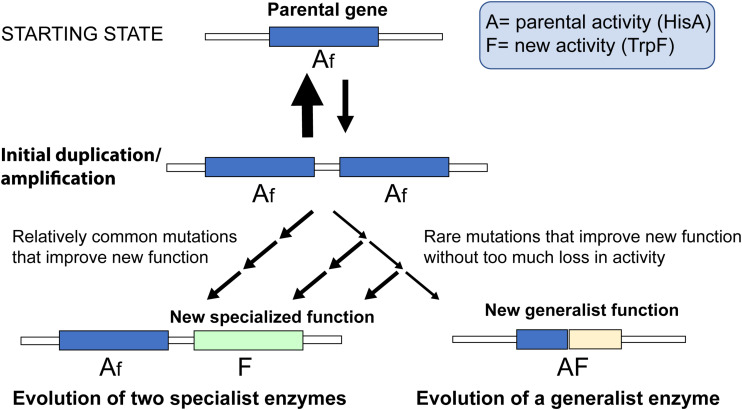
Schematic cartoon of two competing processes. A parental gene with function “A” possesses a minor new function “f.” Selection for increased expression of the new function leads to accumulation of duplications and amplifications of the gene. If the mutational paths are dominated by strong trade-offs, mutations that improve the new function with collateral loss of the parental function are more common compared to mutations that do not lead to large losses in the parental function. Thus, for every mutation that improves the new function the likelihood of retaining the parental function is reduced, and evolution of two specialized enzymes is the more likely outcome than evolution of a single generalist enzyme.

The evolution of a novel enzyme function will depend on the mutation supply and selection (i.e., the ecological context) acting together on a whole organism. In this study, we focus entirely on the mutational aspects and which mutations are available for selection to act on. In Figure 5, we have schematically outlined the two pathways and which factors would influence their relative rates. Since duplication/amplification frequencies in bacterial genomes (and other organisms as well) is about 10^4^- to 10^7^-fold higher than for a typical point mutation (Andersson and Hughes, 2009) it is very likely that the first step for both pathways is a gene amplification that increases the gene dosage of the function that is rate-limiting for growth (in this case TrpF activity). Two key factors will then influence which pathway dominates.

The first factor is the relative frequencies and contributions to activity of the mutations that can increase the TrpF activity as compared to those that increase TrpF activity while retaining some HisA activity. In this study, we identified 16 first-step mutations that resulted in TrpF activity. Of these 16 mutations, 13 resulted in complete loss of HisA activity whereas three maintained some weak activity. In the 2nd- and 3rd-step mutants several additional mutations could confer an increase in TrpF activity but they were all associated with a complete loss of HisA activity. These numbers indicate that the number of available mutations that confer TrpF activity while retaining HisA is very small compared to mutations that can provide TrpF activity (irrespective of what happens with HisA activity). Furthermore, in our previous study, we could identify (after 3000 generations of evolution) only very few generalist enzyme variants that provided substantial activities of both HisA and TrpF.

The second factor is the shape of the trade-off curve between the two functions at each intermediate mutational state. I.e., if any new mutation that improves TrpF activity is associated with a substantial loss of HisA activity that variant is improbable to end up as a bi-functional enzyme. As shown here, there is generally a very strong, reciprocal trade-off between the two functions. From these considerations, we conclude that for the HisA-TrpF experimental system evolution, the process of duplication – divergence (specialization) seems more likely to dominate compared to evolution of a generalist enzyme, at least under conditions where gene duplications are stabilized by selection for the new activity.

## Conclusion

In summary, our data shows that (i) there exists a number of different mutations and mutational pathways toward increased TrpF activity, (ii) that these pathways are associated with a severe loss of the original HisA activity, that (iii) certain mutations occur in several independent lineages and appear to be able to increase TrpF activity on their own, irrespective of the mutational context, whereas other mutations only confer their effect in certain backgrounds and (iv) that the majority of amino acid substitutions that confer the new TrpF activity are located in/near the substrate binding site.

While weak trade-offs with little loss of the original activity during the first steps toward a new activity have been demonstrated in several experimental systems (Chelliserrykattil et al., 2001; Matsumura and Ellington, 2001; Aharoni et al., 2005; Khersonsky and Tawfik, 2010; Rockah-Shmuel and Tawfik, 2012; Guo et al., 2014; Devamani et al., 2016), we show here for HisA that evolution toward TrpF activity is associated with strong trade-offs, and that a majority of the pathways toward increased new activity are associated with complete loss of the original activity.

Furthermore, the measured effects of the mutations on HisA/TrpF activity were dependent on expression levels, implying that high expression levels could partly buffer the deleterious effects on the original activity. Thus, apart from any structural trade-offs at the active site, the ability of the cell to up-regulate the level of protein expression, and thereby increase buffering, is likely to impact the shape of the trade-off curve and the available paths toward increased activity. It is expected that strong trade-offs and poor buffering would promote duplication-divergence pathways since gene duplication would resolve the trade-off problem by maintaining a wild-type *hisA* gene copy, whilst allowing another copy freedom to evolve toward the new TrpF activity. This is in accordance with previous work, showing that long-term evolution to improve both HisA and TrpF activities resulted in most evolving lineages in duplication and divergence and evolution of single-substrate enzymes that performed either HisA or TrpF reactions (Näsvall et al., 2012). Conversely, weak trade-offs and good buffering (by up-regulation of expression) would promote generation of generalist enzymes as the original activity could be retained for a longer time, allowing for the evolution of efficient bi-functional versions of the enzyme. It is notable that in some bacterial species (e.g., *Streptomyces coelicolor* and *Mycobacterium tuberculosis*) a single enzyme, PriA, performs both functions. This bi-functional enzyme is thought to have evolved from *hisA* after loss of the *trpF* gene (Plach et al., 2016). It is known that Streptomycetes do not regulate their amino acid biosynthesis (Hodgson, 2000) and it is possible that constitutive high-level expression of HisA could have buffered any loss of activity associated with evolution of bi-functionality in these bacteria.

There are several caveats associated with this study. First, the use of error-prone PCR to generate mutations obviously does not represent natural mutagenesis and it is possible that biases in the mutation supply are introduced. Second, many possible paths for improving TrpF activity were likely missed as we in each step selected only the mutants that grew visibly faster than the parent from the preceding step and smaller step mutants (that potentially conferred a weaker trade-off) would not have been detected. Third, there might exist multi-mutational pathways toward TrpF activity that would require saltational changes (i.e., many mutations, rearrangement) and that would not be generated by error-prone PCR. Fourth, and most importantly, in our simplified system factors including which selection pressures are present and what are their relative strength and timing are not considered. Thus, whether evolution of a new function proceeds via duplication – divergence or by evolution of a generalist is not only determined by the mutational pathways and trade-offs between new and old function (as studied here), but ecological factors are essential as well (Kassen, 2019).

## Materials and Methods

### Strains and Media

All strains in this study originate from *S. enterica* serovar Typhimurium strain LT2 (DA6192) and are listed in Supplementary Tables S1–S8 together with relative growth rates (as a proxy of total cellular enzyme activity) and their mutations. Lysogeny broth [LB; 5 g/L yeast extract (Oxoid), 10 g/L Tryptone (Oxoid), 10 g/L NaCl (VWR), 1 mM NaOH; Bertani, 1951], and SOC (Hanahan, 1983) were used during construction and re-construction of all strains. LB Agar (LA; LB supplemented with 1.5% [w/v] agar [Oxoid]) was used as solid growth media. LA without NaCl, supplemented with 5% (w/v) sucrose when appropriate, were used for calculating number of transformants and selecting for transformants of reconstructed mutants. M9 minimal media (Miller, 1992) was used for growth rate experiments, and was supplemented with 1.5% (w/v) agar to make solid M9 medium when required. When appropriate, LB media was supplemented with 12.5 μg/ml chloramphenicol or 7.5 μg/ml tetracycline, and M9 minimal media was supplemented with 0.2% (w/v) glucose, 0.2% (v/v) glycerol, 0.1 mM L-tryptophan or 0.1 mM L-histidine. All strains were grown overnight in LB, mixed with dimethyl sulfoxide (DMSO) at a final concentration of 10% (v/v), and frozen at −80°C for long term storage.

### Strain Construction

Phusion High-Fidelity DNA Polymerase (Thermo Fisher Scientific Inc.) was used for PCR amplification of DNA cassettes for constructions and re-constructions through recombineering. Mutagenesis by error prone PCR was performed with GeneMorph II Random Mutagenesis Kit (Agilent Technologies). This mutagenesis method has very little bias in mutation spectrum and results in approximately the same number of transitions and transversions (Rasila et al., 2009; Lundin et al., 2018). DreamTaq DNA Polymerase (Thermo Fisher Scientific Inc.) was used for PCR screening and resultant PCR products used for Sanger sequencing (by Eurofins Scientific). In cases when an isolated *hisA* clone providing TrpF activity had multiple mutations and none of these mutations were observed as the sole mutation in *hisA* within any engineered clone, the single mutant was reconstructed by gene synthesis by gBlock Gene Fragments (Integrated DNA Technologies). The gBlocks were PCR amplified using primers carrying 40 bp overhangs homologous to each end of the of the *ble-sacB* cassette in the *cobA*::*cat-P_*tac*_*-[*ble-sacB*]-*SYFP2* construct. They were then transformed into λ-red competent cells lacking the *hisA* and *trpF* genes and carrying the *cobA*::*cat-P_*tac*_*-[*ble-sacB*]-*SYFP2* construct [DA33713; Lundin et al. (2018)]. The PCR product replaced the *ble-sacB* cassette and transformants were selected for by sucrose resistance. Colonies were screened by PCR and verified by sequencing.

For each transformation, the majority of the cells were plated on selective plates. A fraction of the cells was plated on LA lacking NaCl. The plates were incubated for 24 h and replica plated onto LA lacking NaCl supplemented with sucrose. After 24 h, the plates were put on a UVP Visi-Blue^TM^ Transilluminator and the number of yellow fluorescent colonies was counted. Provided the dilution factor, the number of transformants were calculated.

The 12 variants used as starting strains for continued evolution were also placed under control of the L-arabinose inducible *araBAD* promoter (*P*_*araBAD*_). The genes were amplified with primers carrying 40 bp overhangs, homologous to each end of the *araBAD* operon and electroporated into λ-red competent cells with *hisA* and *trpF* deletions, *P*_*araE*_ replaced with a constitutive *P_*J*__23106_* promoter (Näsvall, 2017) and a *cat-sacB* cassette [*Acatsac1*; GenBank accession number MF124798; (Näsvall, 2017)] in the *araBAD* operon. Successful recombinants were selected by sucrose resistance (i.e., selection for *hisA* replacing the *cat-sacB* cassette).

For assessment of expression levels, a *hisA-rfp* fusion protein was constructed by gene synthesis of gBlock Gene Fragments. Basepairs 100-741 of *hisA* were replaced with a flexible linker and the *rfp* (mScarlet; Bindels et al., 2017) coding sequence (Supplementary Figure S4A).

### Growth Rate Measurements

For growth rate measurements with *hisA* expressed from *P*_*tac*_, M9 minimal media was supplemented with glucose. Upon expression from *P*_*araBAD*_, M9 minimal media was supplemented with glycerol, and 0.05 or 0.0001% L-arabinose. In addition, M9 minimal media was also supplemented with L-tryptophan for HisA activity measurements, and with L-histidine for TrpF activity measurements. Overnight cultures preceding growth rate measurements were supplemented with both L-histidine and L-tryptophan.

Presence or lack of activity was determined by growth on minimal media agar plates for 2 weeks unless otherwise stated. Relative growth rates were used as a proxy for total cellular HisA/TrpF activity (specific activity × concentration). Liquid M9 minimal media was supplemented with histidine (when TrpF activity was assessed) or tryptophan (when HisA activity was assessed).

For growth rate measurements, all strains were streaked on LA plates from the −80°C freezer and incubated overnight at 37°C. Four independent colonies were picked for inoculation in M9 minimal media with appropriate supplements, and allowed to grow overnight at 37°C with shaking (at 195 r.p.m. unless otherwise stated). The cultures were diluted 1:1000 into M9 minimal media with appropriate supplements. 300 μl were added in two separate wells of a honeycomb plate and the cultures were grown in a Bioscreen C Analyzer (Oy Growth Curves Ab Ltd., Helsinki, Finland) at 37°C with shaking until all strains had reached stationary phase or no growth was observed for 4 days. The optical density at 600 nm (OD_600_) was measured every 4 min and exponential growth was observed at 0.02 ≤ OD_600_ ≤ 0.055. When assessing HisA activity, growth rates were measured relative to an isogenic strain carrying a wild-type copy of *hisA* that was grown in the same conditions and when TrpF activity was assessed, growth rates were determined relative to a *S. enterica* wild-type (DA6192). Growth rates (*k*, min^–1^) were calculated by fitting the curve *N*_*t*_ = *N*_0_e^*kt*^, where *t* (min) is time, to the data. Relative growth rates were obtained by dividing the growth rate of each replicate with the average value of all wild-type replicates. If the growth rate of one replicate deviated from the other replicates it was removed and if four or more replicates deviated, the experiment was repeated. The variation within experiments was calculated as the standard deviation of all included replicates. If no growth could be observed after 4 days in liquid minimal media but growth on solid minimal media within 2 weeks was observed, the strains were concluded to have a relative growth rate higher than 0 (i.e., viable on solid minimal media) but lower than 0.05 (lowest *k*, relative to DA6192, observed in any growth rate experiment in this study (data not shown).

### Expression Levels

Eight independent colonies were picked for inoculation of M9 minimal media supplemented with histidine and tryptophan and were allowed to grow overnight. For measurements of expression from the *P*_*tac*_ promoter, the minimal media was also supplemented with glucose and for measurements from the *P*_*araBAD*_ promoter the media was also supplemented with glycerol and 0.05 or 0.0001% L-arabinose. The cultures were diluted 1:1000 in minimal media supplemented with the same components as for the overnight cultures and 300 μl were added to separate wells in a 96-well plate. The cultures were allowed to grow in a Spark multimode microplate reader (Tecan Spark 10 M Trading AG, Männedorf, Switzerland) at 37°C with shaking for 24 h. The optical density at 600 nm wavelength (OD_600_) and the red fluorescence as measured by excitation by monochromator at 570 nm wavelength (bandwidth 20 nm) and emission by filter at 635 nm wavelength (35 nm bandwidth) was measured every 4 min. Growth rates were obtained, outliers removed and measurement variation calculations were performed as previously described.

### Evolution of Lineages

The first step of improvement (1st step improved) was carried out by PCR amplifying (using high-fidelity DNA polymerase) the 12 different starting *hisA* variants and the resulting product was amplified by error-prone PCR using primers carrying 40 bp overhangs homologous to each end of the of the *ble-sacB* cassette in the *cobA*::*cat-P_*tac*_*-[*ble-sacB*]-*SYFP2* construct. The products were transformed into λ-red competent cells lacking the *hisA* and *trpF* genes and carrying the *cobA*::*cat-P_*tac*_*-[*ble-sacB*]-*SYFP2* construct (DA33713). The PCR product replaced the *ble-sacB* cassette and transformants were selected for on M9 minimal media agar supplemented with histidine or tryptophan. The plates were incubated at 37°C for up to 2 weeks or until colonies were visible. The largest colonies were re-streaked on M9 minimal agar, screened by PCR, sequenced, and their fitness was assessed by growth rate measurements as previously described. For the following steps of improvement (2nd and 3rd step improved), colony suspensions (a single colony in 100 μl H_2_O) of all of the clones isolated in the previous step (listed in Supplementary Tables S2, S7) were used as templates in separate error-prone PCR reactions. All PCR products from each lineage were pooled and transformed into λ-red competent cells (DA33713) and transformants were selected and treated as described above for the 1st step improvement. All 3rd step improved clones are listed in Supplementary Table S8.

## Data Availability Statement

The original contributions presented in the study are included in the article/Supplementary Material, further inquiries can be directed to the corresponding author.

## Author Contributions

EL performed the experiments and analyzed the data. All authors planned and designed the experiments and wrote the manuscript.

## Conflict of Interest

The authors declare that the research was conducted in the absence of any commercial or financial relationships that could be construed as a potential conflict of interest.
