# Supplementary material for: Mutational Pathways and Trade-Offs Between HisA and TrpF Functions: Implications for Evolution via Gene Duplication and Divergence
Source: Front Microbiol. 2020 Oct 14;11:588235. doi: 10.3389/fmicb.2020.588235 (PMC7591586; doi:10.3389/fmicb.2020.588235)
Supplement: Supplementary file 1 [file Data_Sheet_1.PDF]

Supplementary Material

Mutational pathways and trade-offs between HisA and TrpF functions: implications for evolution via gene duplication and divergence

Erik Lundin[1], Joakim Näsvall[1] and Dan I. Andersson[1,*]

[1] Department of Medical Biochemistry and Microbiology,
Uppsala University, Sweden

*** Correspondence:**
Dan I. Andersson
dan.Andersson@imbim.uu.se

This PDF file includes:

Figures S1 to S8

Files provided separately:

Supplementary Tables S1 to S5

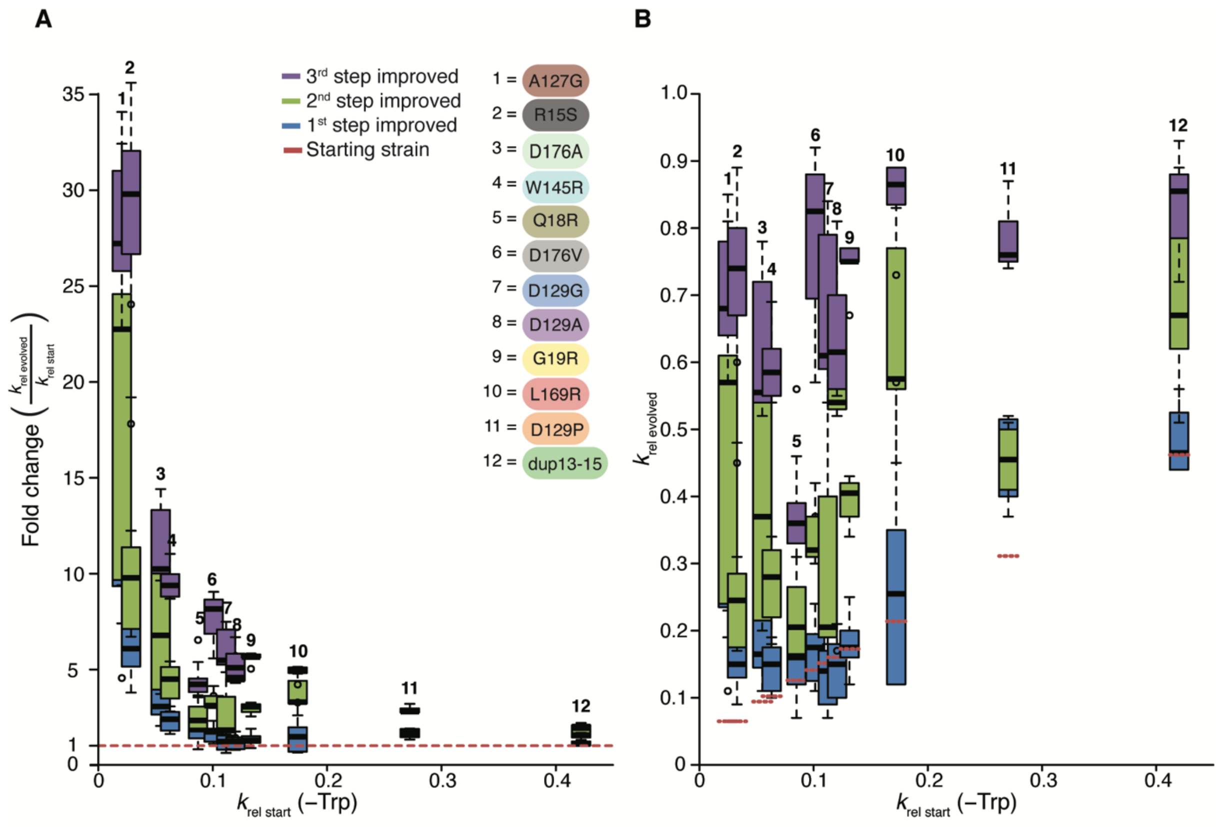

Figure S1. **Boxplots with TrpF activity of improved strains.** Boxplots with relative growth rates (k_{rel}) in medium lacking tryptophan of 1st step (blue boxes), 2nd step (green boxes) and 3rd step (purple boxes) improved strains plotted against the fitness of the starting strain of each lineage. Each box covers 50% of the data, from the first to the third quartile. The middle bar shows the median. Whiskers extend to the most extreme data point which is no more than 1.5 times the interquartile range. Individual dots beyond the whiskers represent outliers. The k_{rel} of the starting strain of the lineage is indicated as dashed red lines. **(A)** Fold change in TrpF activity relative to the starting strain of each lineage ($k_{\text{rel evolved}}/k_{\text{rel start}}$) plotted against TrpF activity of the starting strain ($k_{\text{rel start}}$) of each lineage shows that in general, the lower the starting TrpF activity the higher the relative increase in TrpF activity in all steps of improvement. **(B)** TrpF activity plotted against the activity of the starting strain of each lineage demonstrate the higher the activity of the starting strain, the less room there is for improvements and most lineages, regardless of the starting activity, acquire high activity after three steps of improvements.

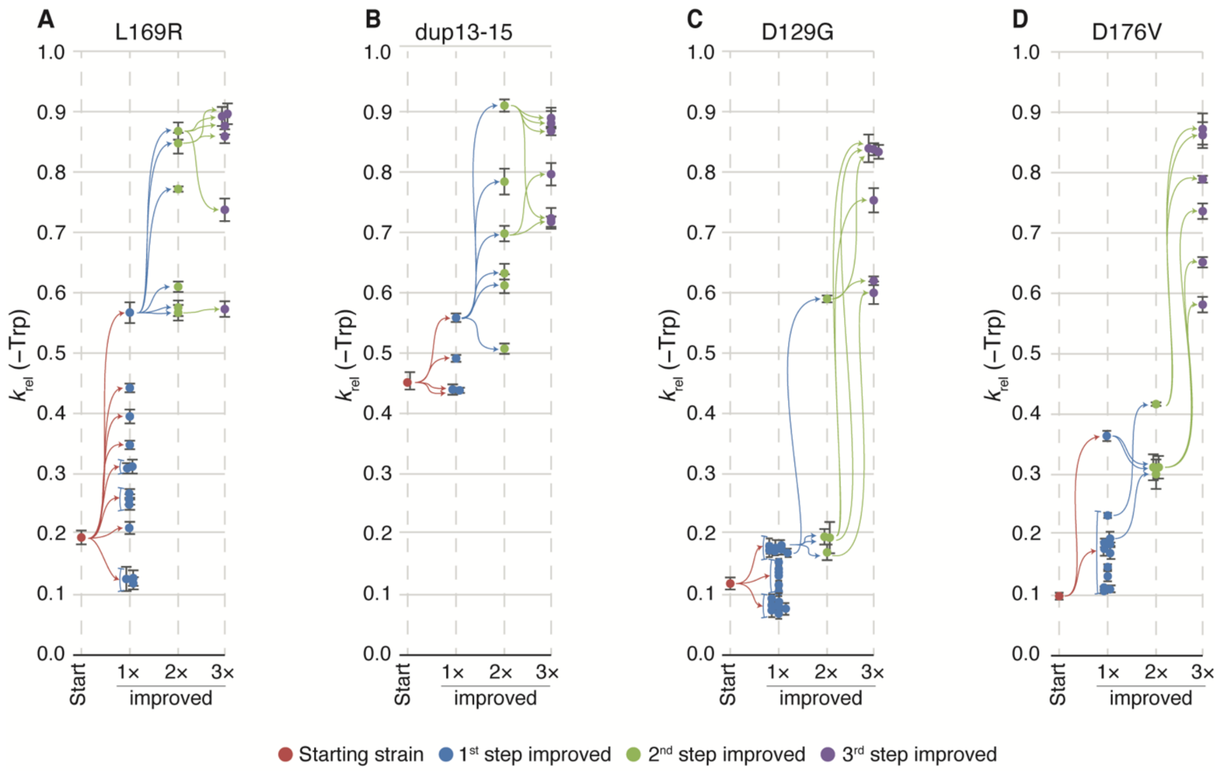

Figure S2. TrpF activity of clones in evolved lineages. To study the reproducibility of the evolution towards increased TrpF activity, the evolution experiment was repeated based on the 1st step improved clones of **(A)** L169R and **(B)** dup13-15 (leading to new 2nd step and 3rd step improved clones) and based on the 2nd step improved clones of **(C)** D129G and **(D)** D176V (leading to new 3rd step improved clones).

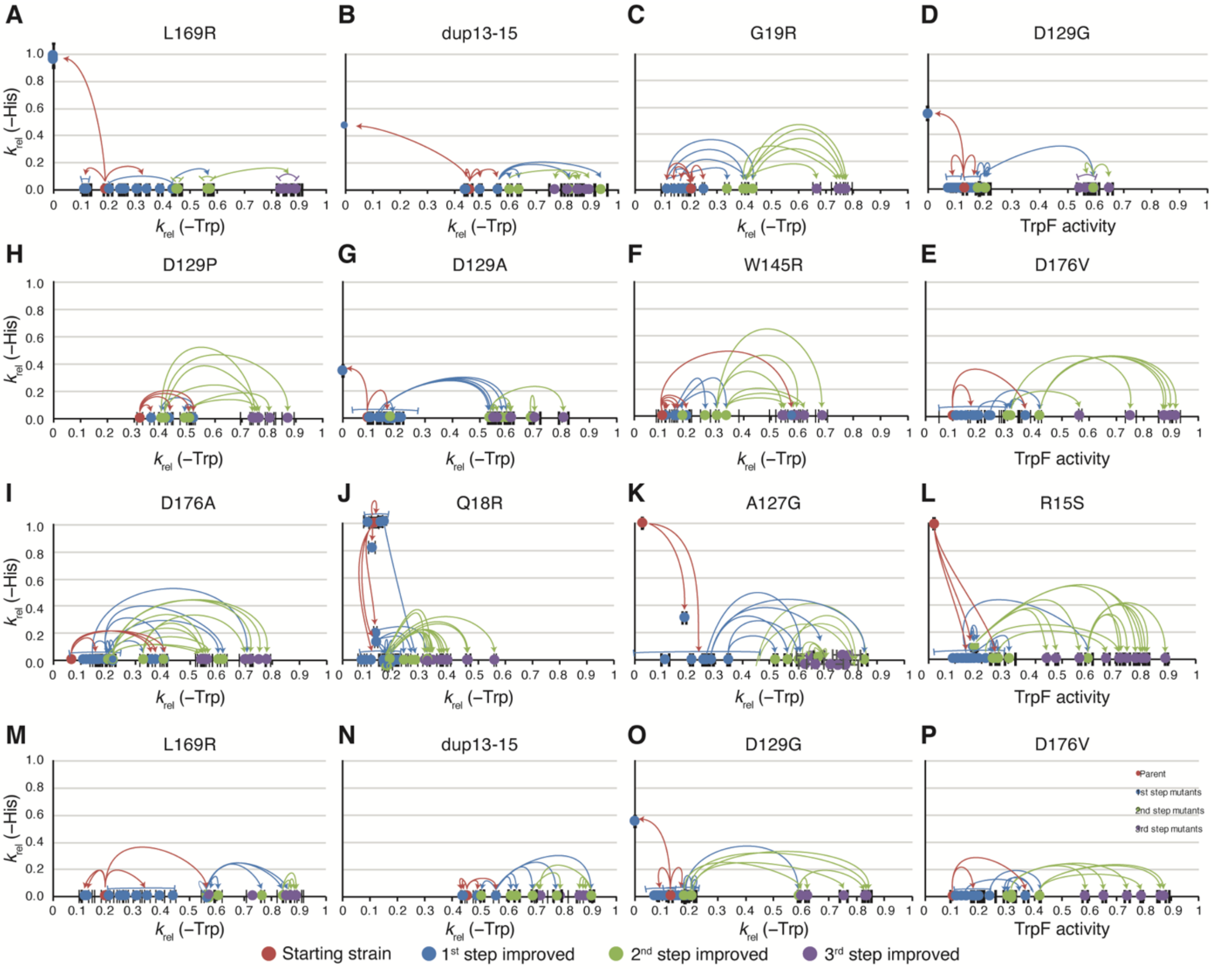

Figure S3. HisA and TrpF activities of clones in evolved lineages. HisA activity was rapidly lost when present in the starting strain and was only possible to introduce into four different lineages with a starting strain lacking HisA activity. Red dots indicate the starting point of each lineage, blue dots represent 1st step improved clones, green represent 2nd step improved clones and, purple represent 3rd step improved clones. Colored arrows indicate that the clone at the end of the arrow is a mutated version of the clone at the beginning of the arrow. Error bars represent the standard deviation of eight replicates. The starting mutation in the 12 lineages evolved towards increased activity were **(A)** L169R, **(B)** dup13-15, **(C)** G19R, **(D)** D129G, **(E)** D129P, **(F)** D129A, **(G)** W145R, **(H)** D176V, **(I)** D176A, **(J)** Q18R, **(K)** A127G and **(L)** R15S.

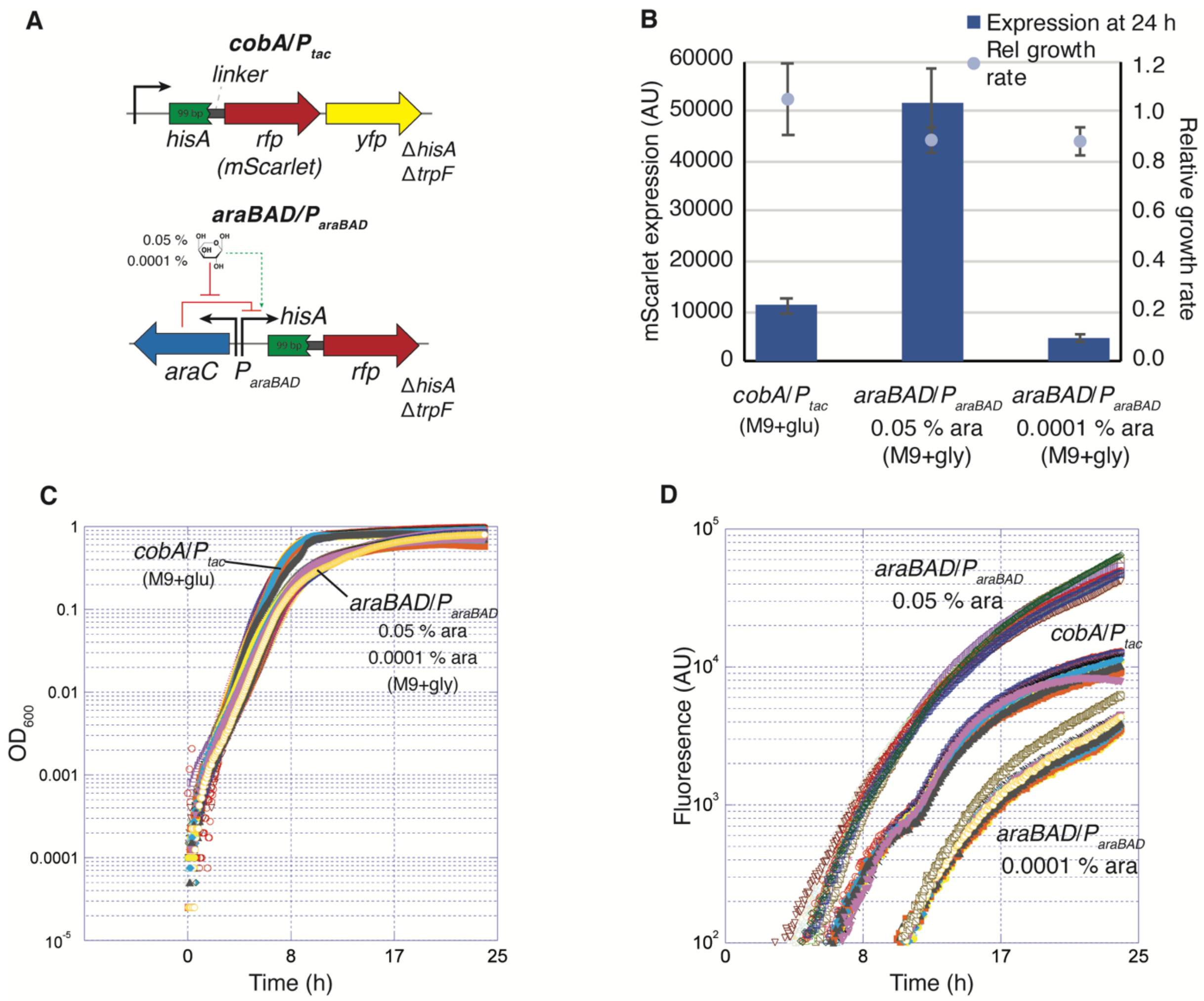

Figure S4. Expression level measurements of constructs with different promoters. Expression levels were assessed at the three different levels used to assess TrpF activity **(A)** Genetic set-up for measuring expression levels of HisA expressed by P_{tac} (*cobA* locus) and P_{araBAD} (*araBAD* locus). (B) Expression levels at 24 h. Expression from P_{araBAD} was studied by inducing with 0.05 % and 0.0001 % L-arabinose. **(B)** *hisA* expression levels after 24 h. **(C)** Exponential growth of *hisA-rfp* fusion expressed from *cobA/*P_{tac} and *araBAD/*P_{araBAD} **(D)** Expression levels over time for the three tested conditions.

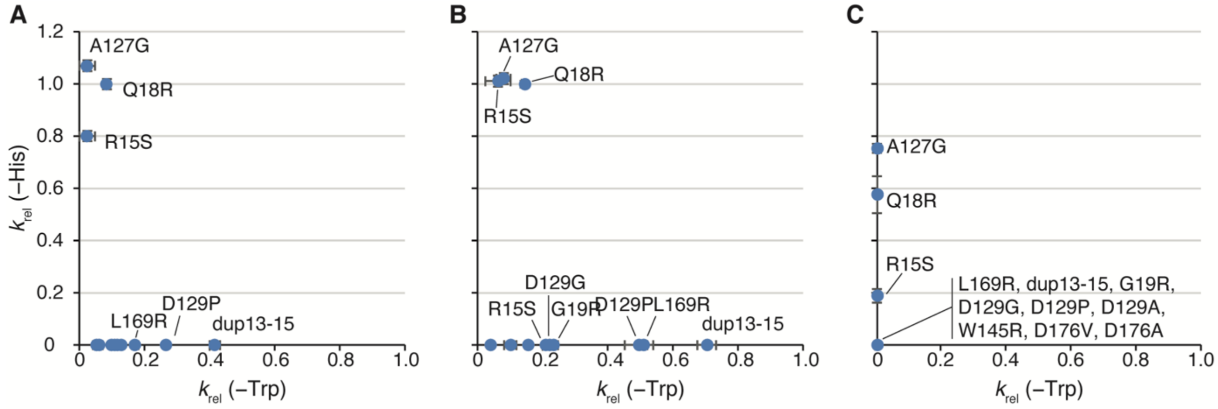

Figure S5. Fitness of HisA mutants with TrpF activity at different expression level. The fitness of HisA with regards to HisA and TrpF activity was measured at three different expression levels. Activities were measured as relative growth rate and error bars indicate standard deviation. HisA activity was measured relative an isogenic strain with *hisA* wild-type and TrpF activity was measured relative an *S. enterica* wild-type. **(A)** HisA and TrpF activities were measured with *hisA* in *cobA* expressed from a P_{tac} promoter (set-up used for continued evolution), **(B)** in *araBAD* with maximum expression (0.05 % arabinose) and **(C)** limiting expression (0.0001 % arabinose) from the P_{araBAD} promoter. With limited expression from the P_{araBAD} promoter, the expression of wild-type *hisA* is limiting for growth.

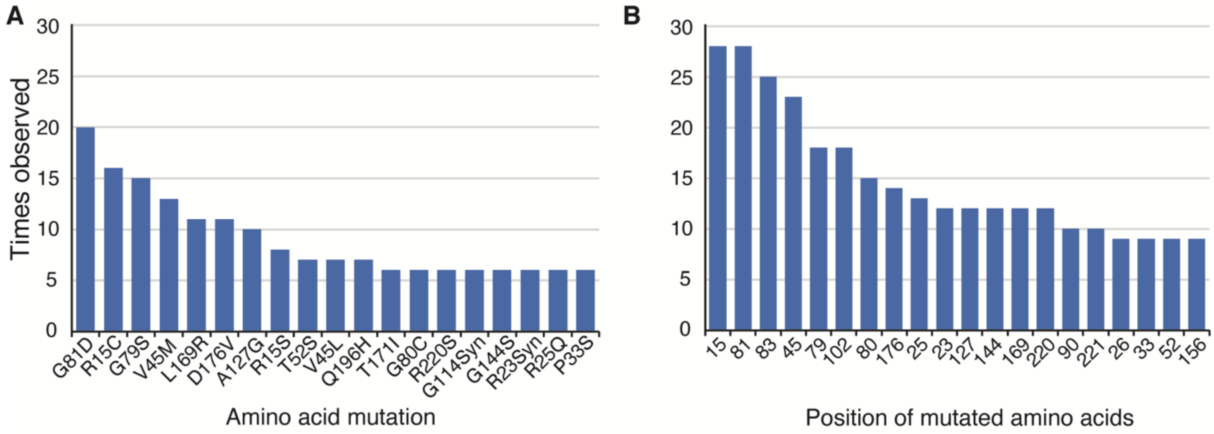

Figure S6. The most frequently observed mutations. Some positions were more frequently mutated than others. **(A)** 18 different mutations were found six times or more in the dataset. **(B)** 20 different positions were mutated nine times or more.

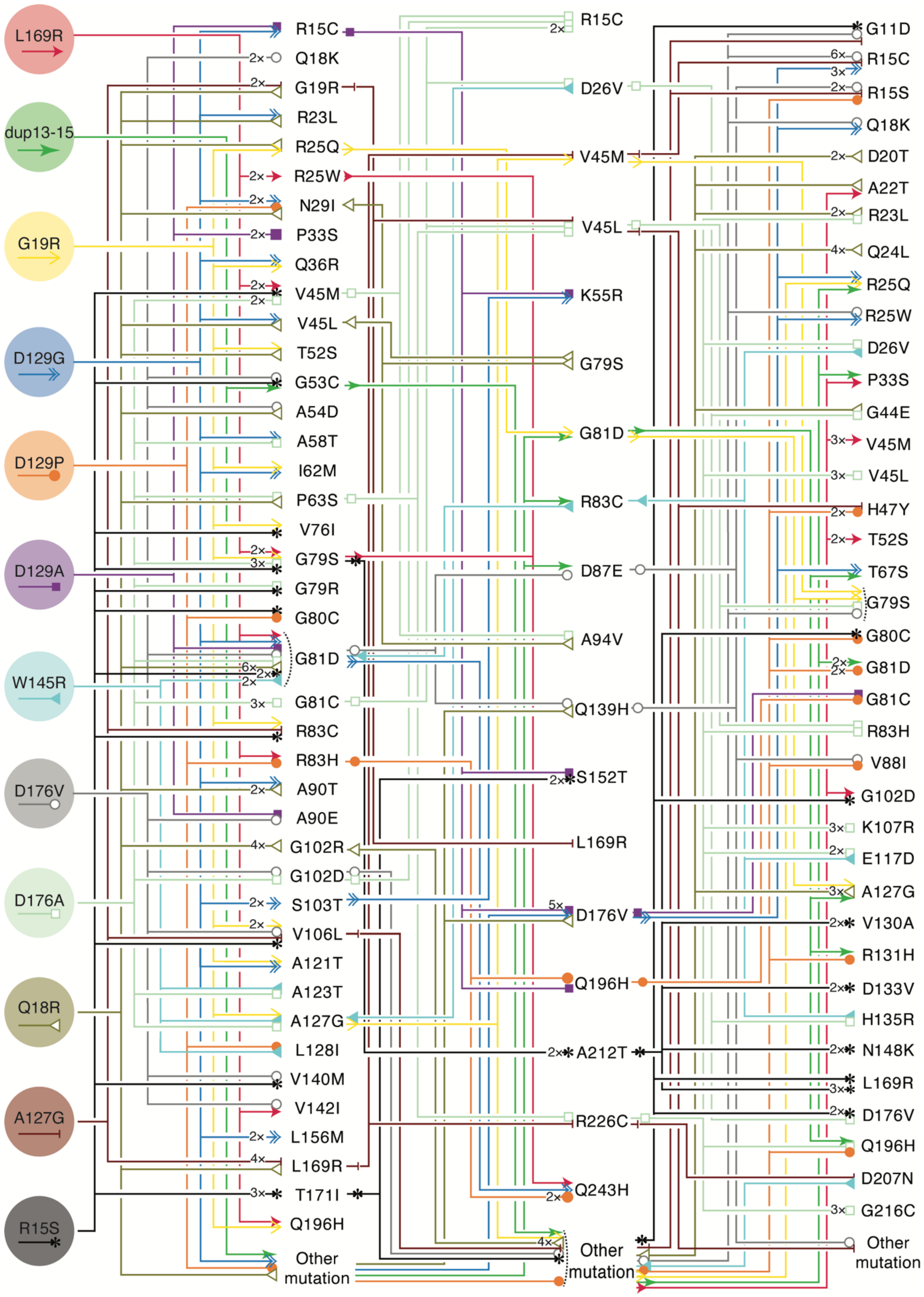

L169R
dup13-15
G19R
D129G
D129P
D129A
W145R
D176V
D176A
Q18R
A127G
R15S
R15C
Q18K
G19R
R23L
R25Q
R25W
N29I
P33S
Q36R
V45M
V45L
T52S
G53C
A54D
A58T
I62M
P63S
V76I
G79S
G79R
G80C
G81D
G81C
R83C
R83H
A90T
A90E
G102R
G102D
S103T
V106L
A121T
A123T
A127G
L128I
V140M
V142I
L156M
L169R
T171I
Q196H
Other mutation
R15C
D26V
V45M
V45L
K55R
G79S
G81D
R83C
D87E
A94V
Q139H
S152T
L169R
D176V
Q196H
A212T
R226C
Q243H
Other mutation
G11D
R15C
R15S
Q18K
D20T
A22T
R23L
Q24L
R25Q
R25W
D26V
P33S
G44E
V45M
V45L
H47Y
T52S
T67S
G79S
G80C
G81D
G81C
R83H
V88I
G102D
K107R
E117D
A127G
V130A
R131H
D133V
H135R
N148K
L169R
D176V
Q196H
D207N
G216C
Other mutation

Figure S7. Mutational pathways towards increased TrpF activity. Twelve different mutants were chosen as starting point for evolution towards increased TrpF activity (first column). After one round of EP-PCR (1st step improved), strains were sequenced and additional mutations occurring two times or more are given in the second column. A path between a starting point mutation (lineage) and a mutation in the second column (1st step improved) indicates that the mutation in the 1st step improved clone was observed in one or several clones from that lineage. Several end points (i.e. mutations in the second column) indicates that several mutations were observed among different clones or within the same clone. 2×, 6× etc. indicates multiple occurrences of a certain mutation among the different clones from the same lineage. A path between a mutation in the second column (1st step improved) and the third column (2nd step improved) indicates that a least one clone among the 2nd step improved clones possessed the mutation from the 1st step improved clones in addition to the starting mutation and the newly acquired mutation. No path from a mutation in the second column (1st step improved) to any mutation in the third column (2nd step improved) indicates that no clones among the 2nd step improved clones contained that mutation from the 1st step improved step. In the same way, a path between a mutation in the third column (2nd step improved) and the forth column (3rd step improved) indicates that at least one 3rd step improved clone acquired a new mutation (forth column, 3rd step improved) in addition to the mutation in the third column (2nd step improved) and all previous mutation. No path from a certain mutation indicates that no clones among the 3rd step improved clones were based on that 2nd step improved clone. All included mutations in each column occur two or more times in the total set of mutation in all lineages in their respective improvement step. “Other mutation” indicates a mutation occurring only once within the total set of mutations of the improvement step (i.e. all lineages having a path through an “other mutation” have unique mutations).

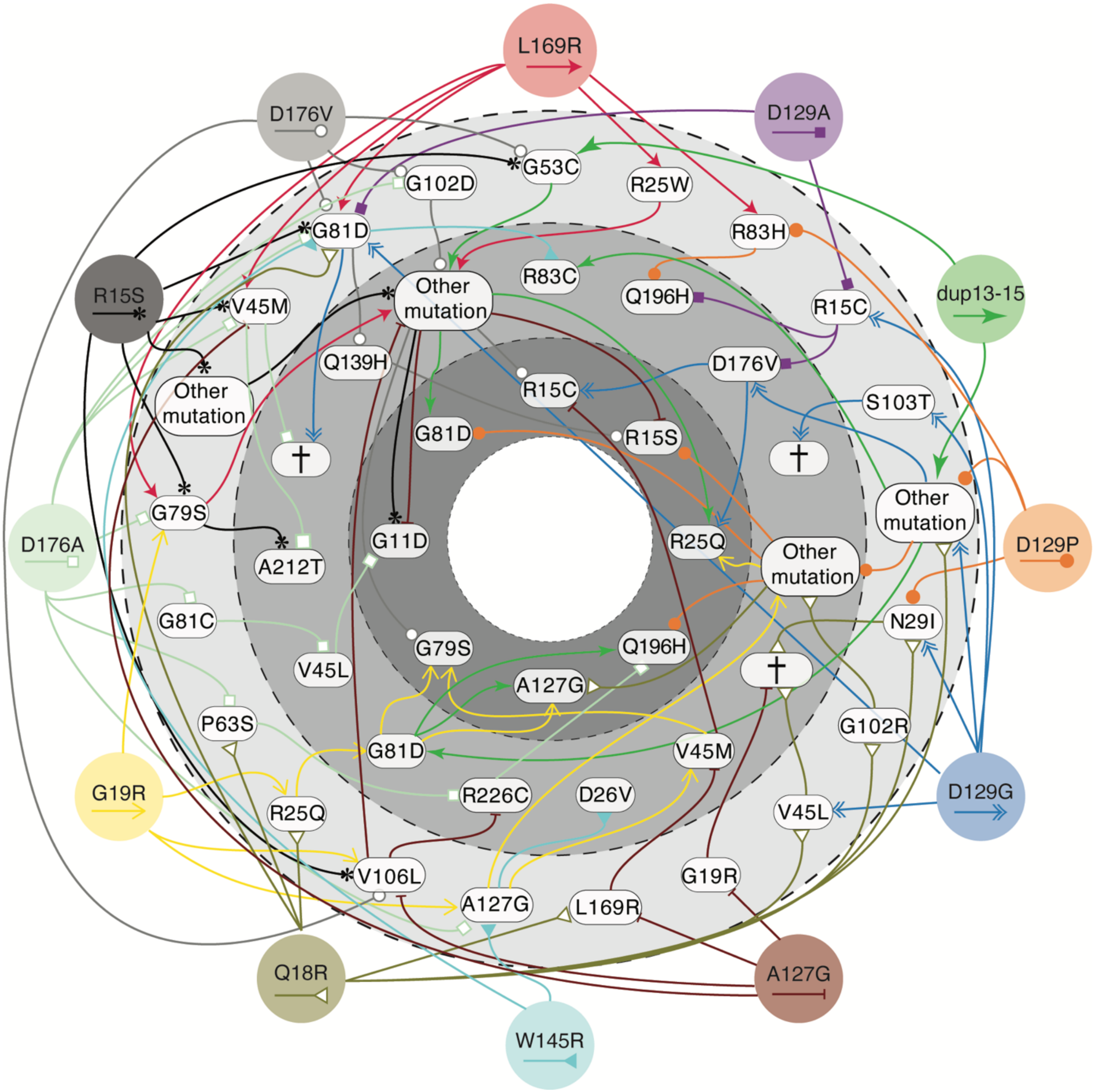

Figure S8. Most common mutational pathways towards increased TrpF activity. The colored circles indicate the starting mutation of each lineage and shows the unique color and symbol given to each lineage. The mutations in the 1st step improved clones that a 2nd step improved clone was based on are given in the outer light grey circle. The mutations in the 2nd step improved clones that a 3rd step improved clone was based on are given in the middle grey circle. The mutations in the 3rd step improved clones that were found in at least three different lineages or at least four times from two different lineages are given in the inner dark grey circle. Arrows between mutations indicate that a clone with the mutation at the end of the arrow (i.e. in a later improvement step) was based on the clone carrying the mutation at the arrow origin. A cross indicates a mutation within the 2nd step improved clones that none of the clones among the 3rd step improved clones carried (i.e. a dead end).
